# CT radiomics model combined with clinical and radiographic features for discriminating peripheral small cell lung cancer from peripheral lung adenocarcinoma

**DOI:** 10.3389/fonc.2023.1157891

**Published:** 2023-03-20

**Authors:** Jingting Wang, Feiyang Zhong, Feng Xiao, Xinyang Dong, Yun Long, Tian Gan, Ting Li, Meiyan Liao

**Affiliations:** ^1^ Department of Radiology, Zhongnan Hospital of Wuhan University, Wuhan, China; ^2^ Department of Radiology, Sir Run Run Shaw Hospital, Zhejiang University School of Medicine, Hangzhou, Zhejiang, China

**Keywords:** small cell lung cancer, lung adenocarcinoma, tomography, X-ray computed, radiomics, diagnostic model

## Abstract

**Purpose:**

Exploring a non-invasive method to accurately differentiate peripheral small cell lung cancer (PSCLC) and peripheral lung adenocarcinoma (PADC) could improve clinical decision-making and prognosis.

**Methods:**

This retrospective study reviewed the clinicopathological and imaging data of lung cancer patients between October 2017 and March 2022. A total of 240 patients were enrolled in this study, including 80 cases diagnosed with PSCLC and 160 with PADC. All patients were randomized in a seven-to-three ratio into the training and validation datasets (170 vs. 70, respectively). The least absolute shrinkage and selection operator regression was employed to generate radiomics features and univariate analysis, followed by multivariate logistic regression to select significant clinical and radiographic factors to generate four models: clinical, radiomics, clinical-radiographic, and clinical-radiographic-radiomics (comprehensive). The Delong test was to compare areas under the receiver operating characteristic curves (AUCs) in the models.

**Results:**

Five clinical-radiographic features and twenty-three selected radiomics features differed significantly in the identification of PSCLC and PADC. The clinical, radiomics, clinical-radiographic and comprehensive models demonstrated AUCs of 0.8960, 0.8356, 0.9396, and 0.9671 in the validation set, with the comprehensive model having better discernment than the clinical model (P=0.036), the radiomics model (P=0.006) and the clinical–radiographic model (P=0.049).

**Conclusions:**

The proposed model combining clinical data, radiographic characteristics and radiomics features could accurately distinguish PSCLC from PADC, thus providing a potential non-invasive method to help clinicians improve treatment decisions.

## 1 Introduction

Lung cancer is the leading cause of cancer mortality worldwide in both men and women (1, 2) and is classified into two histological subtypes: small cell lung cancer (SCLC) and non-small cell lung cancer (NSCLC), with adenocarcinoma (ADC) being one of the most common types of the latter (3). Depending on the location of primary lesions, lung cancers could be divided into central and peripheral types. The central type of lung cancer is mostly squamous-cell carcinoma and SCLC, while the peripheral type is mainly ADC (4–6). Peripheral SCLC (PSCLC) is relatively less common in clinical practice, accounting for 15-30% of all lung cancers (6, 7). PSCLC typically originates in the bronchial submucosa and infiltrates into the peribronchial connective tissue, resulting in bronchial stenosis being less likely to occur in its early stage. PSCLC is easy to overlook due to its non-obvious clinical symptoms and low diagnostic rate (7, 8). The tumors are already extensively metastasized by the time of detection, leading to most patients not benefiting from surgery or local radiotherapy (8). PSCLC is more aggressive and malignant than peripheral ADC (PADC) and they have significant differences in responses to therapy. Surgical resection is preferred for early-stage ADC. The high prevalence of surgery and the rapid development of targeted therapies, such as individualized treatment based on specific histological and molecular subtypes, have greatly improved the survival time of NSCLC, especially ADC (9, 10). However, extreme caution is required in SCLC surgical decision-making. Surgery is only recommended for certain patients with surgically resectable stage I to IIA SCLC (11). SCLC is highly sensitive to initial chemotherapy and radiotherapy. Postoperative adjuvant radiotherapy could significantly improve the 5-year survival rate (9). In addition, although radiotherapy is the most available treatment for patients with middle-late stage SCLC or NSCLC, there are significant differences between the two treatment regimens. Therefore, early, rapid, and accurate differential diagnosis of SCLC and NSCLC plays a crucial role in the treatment decisions and can improve the survival rate and prognosis of patients.

Pathological biopsy is still the gold standard for diagnosis of lung cancer. However, the NCCN Clinical Practice Guidelines in Oncology also suggest that patients with a strong clinical suspicion of stage I or II lung cancer (based on risk factors and radiologic appearance) do not require a biopsy before surgery. The biopsy adds time, costs, and procedural risk and may not be needed for treatment decisions (12). So, the initial determination of the pathological type of lung cancer is needed before treatment. Magnetic resonance imaging and computed tomography (CT), are widely used in clinical settings to investigate the anatomy and function of the body in both health and disease (13). CT images, providing qualitative morphological information, have been the preferred choice for lung cancer screening and diagnosis (14). Many studies have found some morphological CT features differed between NSCLC and SCLC (8, 14–16). Nonetheless, PSCLC, especially early-stage PSCLC, remains difficult to diagnose, some of the imaging features lack specificity, overlapping with peripheral NSCLC and other peripheral tumors. Furthermore, traditional imaging diagnostic accuracy was easily affected by the physician experience and subjective factors. Some research has shown that clinical data, particularly serum tumor markers, could be helpful predictors of the pathological type of lung cancer (17). Radiomics, a potentially non-invasive data-mining method, extracts high-throughput features from routinely acquired radiographic medical images (18). The comprehensive and detailed tumor characterization could be reflected by radiomics, which is cost-effective and non-invasive. Several studies related to radiomics showed very positive and promising results in the detection of lung cancer, the prediction of histology and subtypes, the prediction of prognosis and the assessment of treatment outcome (19). However, there are very few studies in the differential diagnosis between PSCLC and PADC.

This study retrospectively analyzed the clinical, radiographic, and radiomics characteristics between PSCLC and PADC patients. The aim of the study was to develop and validate a CT-based radiomics model to classify PSCLC and PADC and to investigate whether the addition of clinical and radiographic factors could improve the performance of the diagnostic model.

## 2 Materials and methods

### 2.1 Study population

We retrospectively identified participants with pathologic analysis–proven lung cancer from October 2017 to March 2022 and collected clinical and imaging data. The inclusion of this study was based on the following criteria: (1) thin-section (≤1.5 mm) CT scan performed within two weeks before needle biopsy or surgery; (2) tumors were located below the lung segment bronchus; and (3) without anti-cancer treatment before CT scan. Patients were excluded according to the following criteria: (1) incomplete image data or poor image quality; (2) ground-glass nodules (GGN); (3) absence of complete clinical data; and (4) with some other kind of primary malignancy. Due to this research’s retrospective nature, a waiver of informed consent for this study was allowed, and the hospital ethics committee approved the study (2021057).

### 2.2 Clinical data

Seven clinical features, including gender, age, history of smoking, the preoperative clinical stage and serum tumor markers [neuron-specific enolase (NSE), carcinoembryonic antigen (CEA), and carbohydrate antigen 125 (CA125)] were collected when reviewing the electronic medical record system. Preoperative clinical stage of lung cancer was based on the tumor/node/metastasis (TNM) classification of malignant tumors, 8th edition. The upper limit of the normal value of various serum tumor markers was used as the positivity threshold, with the following positivity criteria: CEA>5ng/mL, NSE>16.3U/ml, and CA125>35U/mL. The above tumor markers were taken as positive when their values were beyond the upper limit of the normal range. Data extraction was performed by two authors (JW and TG) independently and differences were resolved by a third investigator (FZ).

### 2.3 CT scanning

CT examinations were performed *via* two CT scanners (Somatom Definition 64 and Somatom Sensation 16; Siemens Healthcare, Forchheim, Germany). Technical parameters were: automatic tube current adjustment technology, 100-350 mAs; tube voltage, 120 kV; slice interval, 0 mm; reconstructed section thickness, 1 mm. The original images were reconstructed using a standard soft-tissue algorithm.

### 2.4 Visual assessment of CT signs

**Figure 1:**
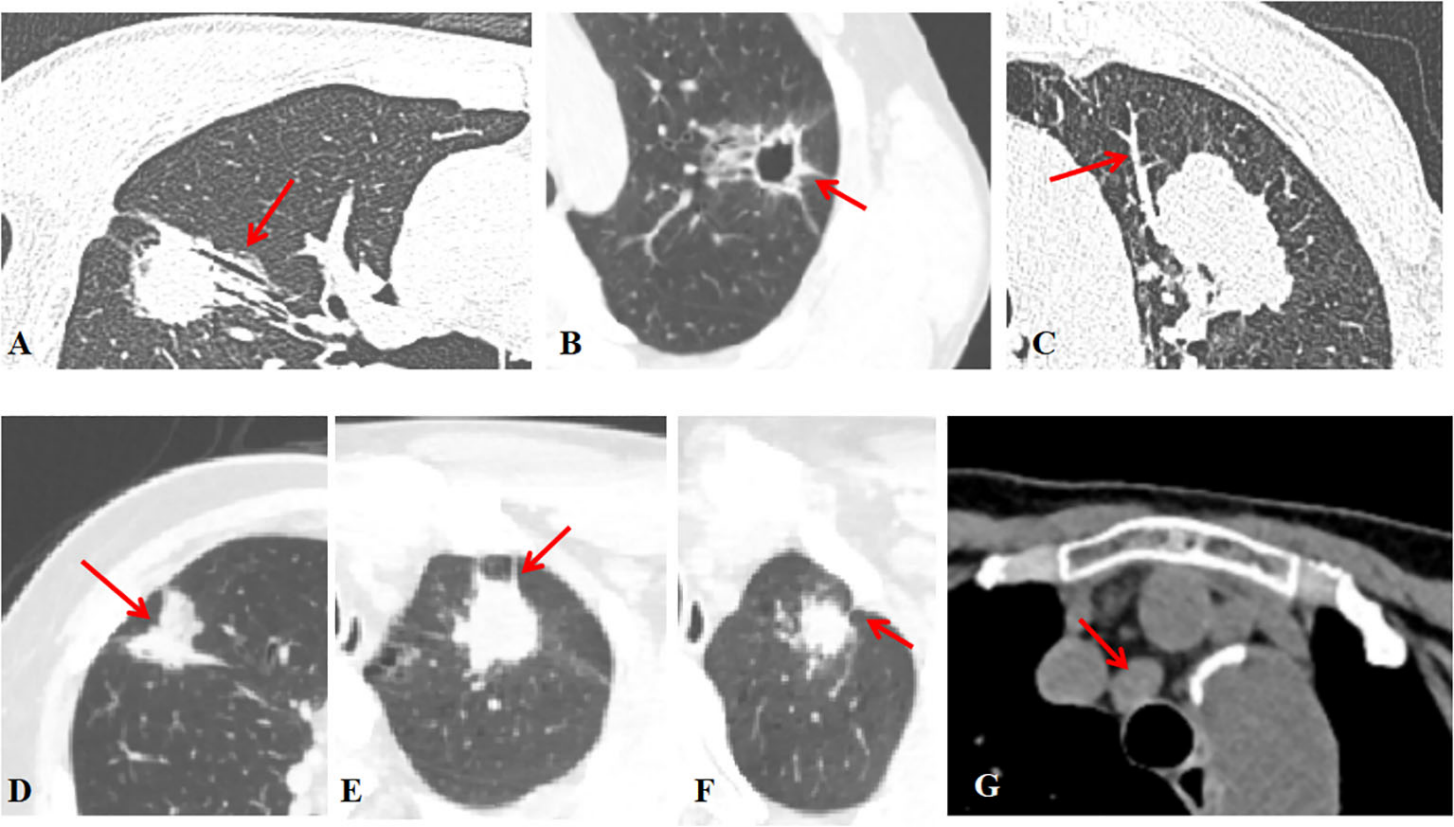
Radiographic features **(A)** The air bronchogram is fluid-filled or solid alveoli surrounded by air-filled bronchus. **(B)** The cavity is a gas-filled space >3 mm within a solid lung lesion, mass or nodule. **(C)** Vessel convergence sign was defined as an increase in internal vessels or clustering of vessels. **(D)** Lobulation was defined as a nodal margin showing an uneven lobar outline. **(E)** Spiculation was defined as a small spine-like protrusion from the nodule margin extending into the lung parenchyma without reaching the pleural surface. **(F)** Pleural indentation was defined as a tapered or linear extension of the lesion into the pleura, reflecting pulmonary fibrosis with retraction of the adjacent pleura. **(G)** Hilar and mediastinal lymph nodes were considered positive if the short axis exceeded 10 mm on chest CT images.

The analysis of the CT images was performed at the lung window setting (width, 1500 HU; level, -700 HU), and the following CT signs were analyzed: maximal lesion size, single/multifocal lesion, the specific lung lobes involved, air bronchogram, cavity, calcification, vessel convergence, margin, contour, lobulation, spiculation, pleural indentation, pleural effusion, peripheral emphysema, and enlarged mediastinal lymph node (see **Figure 1** for definitions). Two radiologists (JW and FZ, who had been practicing diagnostic chest imaging for 2 and 5 years, respectively) reviewed CT images separately. Both radiologists were blinded to the clinicopathological information. In case of any discrepancies between the evaluations, a consensus was reached through discussion.

### 2.5 Clinical and radiographic risk factors

Significant clinical and radiographic features were screened out in univariate logistic regression and subsequently analyzed in multivariate logistic regression analysis in the training cohort. The significant variables in the multivariate logistic regression were identified as potential risk factors for the construction of the clinical-radiographic model. The participant cohort was randomly assigned to 2 sets—a training set (70%) and a validation set (30%). The former was used for feature filtering and model construction, while the latter was used for model validation only.

### 2.6 CT images segmentation, feature extraction, and model building

To reduce the variation caused by different CT scanners acquisition parameters, all images were resampled to the same voxel size (1mm*1mm*1mm) with the linear interpolation method (20). The volumes of interest (VOIs) of lesion areas were segmented by a radiologist (a 2-year work experience) semi-automatically with ITK-SNAP software (version 3.8.itksnap.org). Care should be taken to avoid areas of the chest wall, lung parenchyma, great vessels, and bronchi around the mass and to outline the contour of the mass as completely as possible.

Intra-class correlation coefficients (ICC) were adopted to assess inter- and intra-observer agreement and to ensure reproducibility and stability of results. Feature reliability and reproducibility were tested using a cohort of twenty randomly selected samples. The consistency of the extracted features obtained between the first observer (JW) and a second observer (YL) (inter-observer reliability) was explored, as well as the consistency of the extracted features obtained by the exact first observer (JW) at different times (intra-observer reproducibility). The ICC was used to determine the consistency of the extracted features, and intra- and inter-observer variability was analyzed *via* calculating the Dice similarity coefficient (DSC). The DSC between two observers (JW and YL) (**Table S2A**) and between the two observations of the first observer (JW) (**Table S2B**) was 0.87 ± 0.05 and 0.86 ± 0.06, respectively, which was considered to achieve a good agreement (21). The remaining VOIs were delineated by the first radiologist (JW). Radiomics features were extracted from the pre-processed images with AK Software (Artificial Intelligence Kit; GE Healthcare) and used for subsequent modeling. **Supplementary Material** provided further details on feature extraction.

Features were ranked for importance by the minimum redundancy–maximum relevance (mRMR) algorithm. The top-ranked radiomics features were then picked out by the least absolute shrinkage and selection operator (LASSO) to obtain an optimal subset of radiomics features, which was employed to build the radiomics score (rad-score) to identify PSCLC and PADC. Each lesion’s rad-score is calculated based on the linear combination of selected features, weighted by their coefficients. The study grouped the selected features into four models—the clinical model (only with clinical features), the radiomics model (radiomics features), the clinical-radiographic model (selected clinical and radiographic features), and the comprehensive model (radiomics, clinical and radiographic features). The individualized nomogram incorporated the clinical-radiographic characteristics with rad-score, allowing a simple, visual graphical representation of the risk of PSCLC (22).

### 2.7 Performance assessment

The discriminative efficacy from the above models was quantified based on the AUC values of the training and validation sets. Calibration curves were plotted with the Hosmer-Lemeshow test as a calibration indicator. The clinical utility of the nomogram was assessed by the decision curve analysis (DCA), which quantified the potential net effect of applying the model at different threshold probabilities.

### 2.8 Data analysis

All statistical analyses were carried out with SPSS 25.0 and R 4.1 statistical software. For the continuous variables, the Kolmogorov-Smirnov and Levene tests were first performed to verify the normality and cardinality of the samples. Variables conforming to the normal distribution and chi-square measures were expressed as Mean ± SD. Group comparisons were made using the t-test or Wilcoxon rank-sum test. Categorical variables were expressed as frequencies and percentages, and comparisons between groups were made using the χ² test. A two-sided P<0.05 indicated a statistically significant difference.

The “mRMR” and “glmnet” packages were used to conduct the mRMR algorithm and LASSO regression, respectively. The “pROC” package was used to plot the ROC curves and measure AUCs, which were compared using the Delong test. Nomogram was generated using the “rms” package. “irr” was used to calculate the ICC algorithm. The goodness of fit test was examined using “ResourceSelection” and “ggpubr” was used for data result visualization. The “CalibrationCurves” and “DecisionCurve” packages were used for the calibration curves and DCA analyses.

## 3 Results

### 3.1 Patients

885 cases of peripheral lung cancer were collected, including 423 cases of lung ADC, 123 cases of lung SCLC, and 39 cases of lung squamous-cell carcinoma. A total of 240 cases with 218 males and 22 females who met the inclusion/exclusion criteria were identified (mean age 64.14 years, range 34-83 years). **Table 1** listed the baseline characteristics of the research population. In the ratio of 7:3 ratio, 170 patients were assigned to the training set (55 PSCLCs and 115 PADCs), while 70 patients were to the validation set (25 PSCLCs and 45 PADCs).

**Table 1:** Comparison of clinical data in PSCLCs and PADCs.

Variables	SCLC Group (N=80)No. of patient (%)	ADC Group (N=160) No. of patient (%)	P value
Age (years)			0.961
Mean ± SD	64.18 ± 7.86	64.13 ± 7.35	
Gender			0.429
Male	71 (88.75)	147 (91.875)	
Female	9 (11.25)	13 (8.125)	
Smoking history			0.555
Yes	53 (66.25)	112 (70.00)	
No	27 (33.75)	48 (30.00)	
NSE			<0.001
Abnormal	58 (72.50)	20 (12.50)	
Normal	22 (27.50)	140 (87.50)	
CEA			0.014
Abnormal	30 (37.50)	87 (54.375)	
Normal	50 (62.50)	73 (45.673)	
CA125			<0.001
Abnormal	18 (22.50)	75 (46.875)	
Normal	62 (77.50)	85 (53.125)	
Clinical stage			0.521
Early (I, II)	39 (48.75)	71 (44.375)	
Late (III, IV)	41 (51.25)	89 (55.625)	

### 3.2 Clinical and radiographic factors in PSCLCs and PADCs

Univariate analysis of the clinical and radiographic factors showed that serum tumor marker levels, including NSE, CEA, and CA125, as well as the visual assessment of CT signs such as contour, pleural indentation, spiculation, air bronchogram, and lobulation, were significantly related to PSCLC (P<0.05), as shown in **Table 2**.

**Table 2:** Results of univariate analysis for the classification of PSCLC and PADC in the training set.

Variables	SCLC Group (N=55) No. of patient (%)	ADC Group (N=115) No. of patient (%)	P value
Age (years)			
Mean ± SD	63.73 ± 8.25	64.10 ± 7.44	0.766
Gender			0.306
Male	48 (87.3)	106 (92.2)	
Female	7 (12.7)	9 (7.8)	
History of smoking			0.858
Yes	38 (69.1)	81 (70.4)	
No	17 (30.9)	34 (29.6)	
NSE			<0.001
Abnormal	38 (69.09)	16 (13.91)	
Normal	17 (30.91)	99 (86.09)	
CEA			0.004
Abnormal	18 (32.73)	65 (56.52)	
Normal	37 (67.27)	50 (43.48)	
CA125			<0.001
Abnormal	11 (20.0)	54 (46.96)	
Normal	44 (80.0)	61 (53.04)	
Maximal lesion size (mm)			0.675
Mean ± SD	36.40 ± 16.23	35.37 ± 14.19	
Lesion location			0.115
Right upper lobe	16 (29.1)	49 (42.6)	
Right middle lobe	1 (1.8)	6 (5.2)	
Right lower lobe	17 (30.9)	21 (18.2)	
Left upper lobe	9 (16.4)	24 (20.9)	
Left lower lobe	12 (21.8)	15 (13.0)	
Contour			0.003
Round	38 (69.1)	51 (44.3)	
Irregular	17 (30.9)	64 (55.7)	
Lobulation			0.034
Present	21 (38.2)	26 (22.6)	
Absent	34 (61.8)	89 (77.4)	
Spiculation			<0.001
Present	6 (10.9)	100 (87.0)	
Absent	49 (89.1)	15 (13.0)	
Margin			0.115
Clear	47 (85.5)	86 (74.8)	
Unclear	8 (14.5)	29 (25.2)	
Calcification			0.681
Present	15 (27.3)	28 (24.3)	
Absent	40 (72.7)	87 (75.7)	
Cavity			0.085
Present	15 (27.3)	47 (40.9)	
Absent	40 (72.7)	68 (59.1)	
Air bronchogram			0.001
Present	2 (3.6)	29 (25.2)	
Absent	53 (96.4)	86 (74.8)	
Vessel convergence sign			0.07
Present	21 (38.2)	61 (53.0)	
Absent	34 (61.8)	54 (47.0)	
Pleural indentation			<0.001
Present	12 (21.8)	91 (79.1)	
Absent	43 (78.2)	24 (20.9)	
Peripheral emphysema			0.179
Present	31 (56.4)	77 (67.0)	
Absent	24 (43.6)	38 (33.0)	
Enlarged mediastinal lymph node			0.071
Present	34 (61.8)	54 (47.0)	
Absent	21 (38.2)	61 (53.0)	
Single/multifocal lesion			0.716
Multiple	12 (21.8)	28 (24.3)	
Single	43 (78.2)	87 (75.7)	
Pleural effusion			0.786
Present	11 (20.0)	21 (18.3)	
Absent	44 (80.0)	94 (81.7)	
Clinical stage			0.490
I	11 (20.00)	31 (26.96)	
II	16 (29.09)	22 (19.13)	
III	12 (21.82)	26 (22.61)	
IV	16 (29.09)	36 (31.30)	

All statistically significant clinical and radiographic variables in the univariate analysis were further entered into the multivariate logistic regression analysis (**Table 3**). In the multivariate analysis, five variables, including NSE level, CEA level, spiculation, air bronchogram, and pleural indentation, were ultimately selected as predictors associated with PSCLC, and further included in the clinical-radiographic model.

**Table 3:** Results of multivariate logistic regression analysis for classifying PSCLC and PADC in the training set.

Variables	P value	OR (95% CI)
NSE	<0.001	90.372 (8.841-923.732)
CEA	0.019	0.167 (0.037-0.745)
CA125	0.364	0.456 (0.084-2.486)
Contour	0.272	2.462 (0.494-12.272)
Lobulation	0.058	5.524 (0.944-32.343)
Spiculation	<0.001	0.010 (0.001-0.105)
Air bronchogram	0.022	0.016 (0.001-0.556)
Pleural indentation	0.028	0.187 (0.042-0.834)

### 3.3 Feature extraction

**Figure 2:**
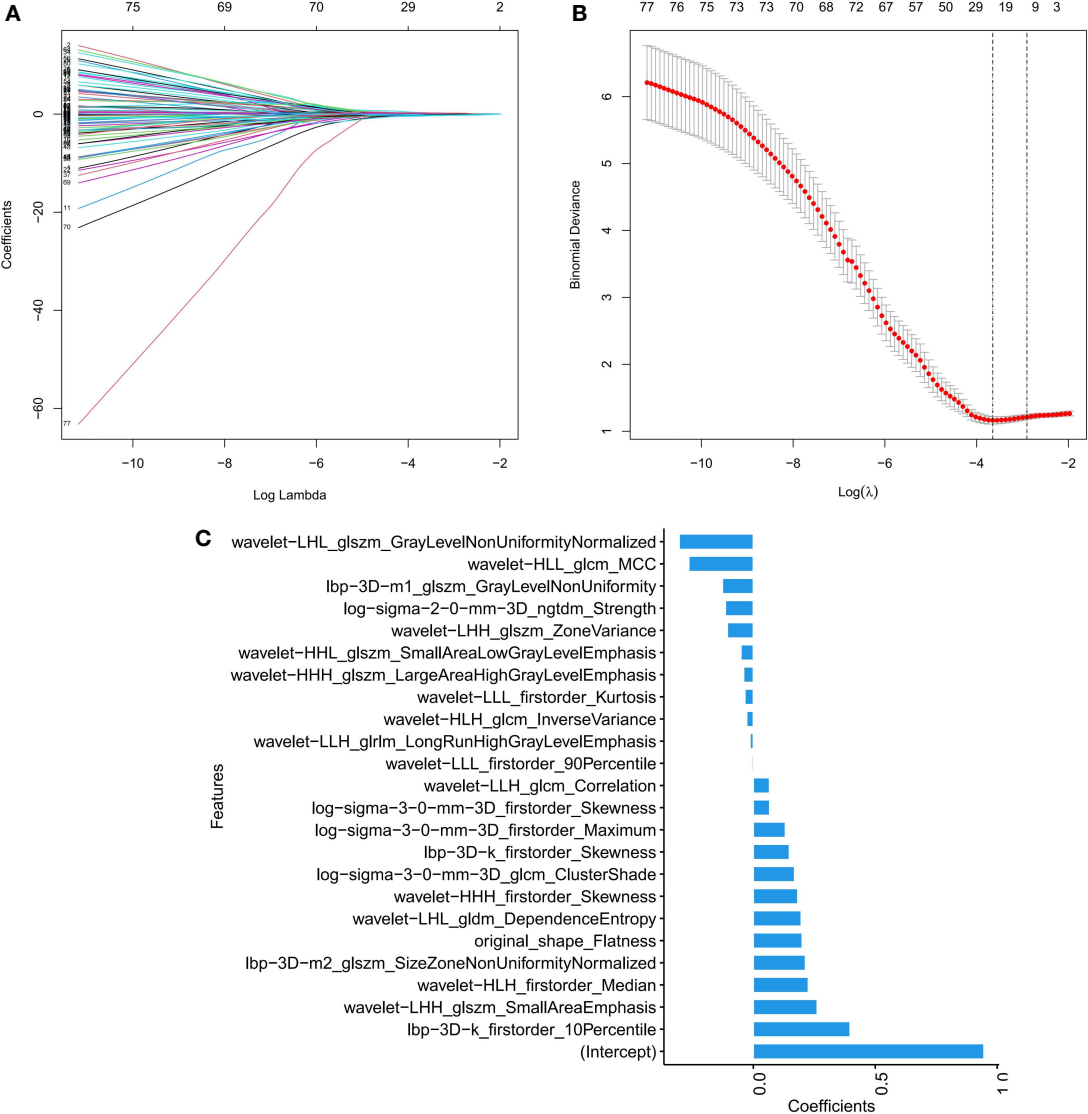
Feature selection by LASSO binary logistic regression model. **(A)** Selecting characterization *via* LASSO regression and 10-fold cross-validation method. **(B)** Coefficient curves based on radiomics features with non-zero coefficients are determined by λ. **(C)** Screening the absolute values of 23 radiomics features and their corresponding coefficients.

**Figure 3:**
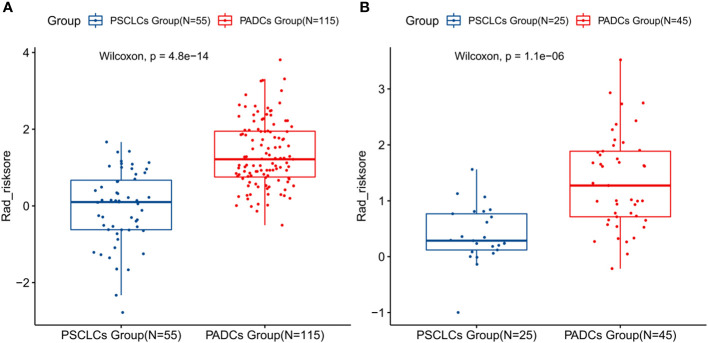
Boxplots presenting the statistical differences between PSCLCs and PADCs in the training set **(A)** and validation set **(B)**.

Among the 1316 radiomics features extracted from CT images, the top 100 mRMR-ranked features were selected for training using the LASSO classifier (23). Finally, based on the retained 23 features, the rad-score formula was derived from the LASSO weighting coefficients, as shown in **Figure 2**, and the rad-score of each lesion was calculated. The rad-score calculation formula was described in the **Supplementary Material**. The rad-score of SCLC in the training set was lower than the rad-score of ADC (-0.044 ± 0.960 vs. 1.348 ± 0.862, P < 0.001), which was confirmed in the validation set (0.393 ± 0.508 vs. 1.348 ± 0.843, P < 0.001), as **Figure 3** showed.

### 3.4 Establishment and validation of models

**Figure 4:**
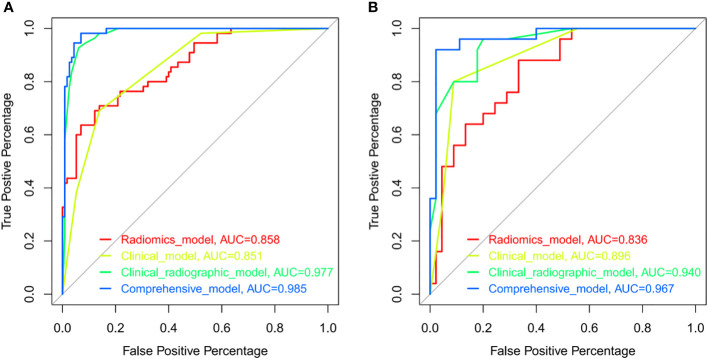
ROCs of the radiomics model (red), clinical model (yellow), clinical-radiographic model (green), and comprehensive model (blue) in the training **(A)** and validation **(B)** sets. ROC indicated the differential diagnostic efficacy of each model. The more accurate the diagnostic model, the more the ROC shifts toward the Y-axis, with the AUC approaching a value of 1.

We constructed the radiomics model based on the rad-score and the clinical-radiographic model using five clinical and radiographic features, which were screened by the multivariate analysis, including NSE level, CEA level, spiculation, pleural indentation, and air bronchogram. The clinical model was constructed with the selected clinical features including NSE level and CEA level. The comprehensive model incorporated significant clinical and radiographic factors with the radiomics signature of CT images. The AUCs of the radiomics model, clinical model, clinical-radiographic model, comprehensive model was 0.8579 (95% CI, 0.7988-0.917), 0.851 (95% CI, 0.7956-0.9054), 0.9774 (95% CI, 0.9579-0.9969) and 0.9851 (95% CI, 0.9702-0.9999) in the training sets (**Figure 4A**) and 0.8356 (95% CI, 0.7419-0.9292), 0.8964 (95% CI, 0.8262-0.9667), 0.9396 (95% CI, 0.8867-0.9924) and 0.9671 (95% CI, 0.9251-0.9999) in the validation sets (**Figure 4B**), respectively. The comprehensive model outperformed the radiomics model (Z=-2.7472, P=0.006), the clinical model (Z=-2.0916, P=0.036) and the clinical-radiographic model (Z=-1.9639, P=0.049) according to the Delong test, which was statistically significant in the validation cohort.

**Figure 5:**
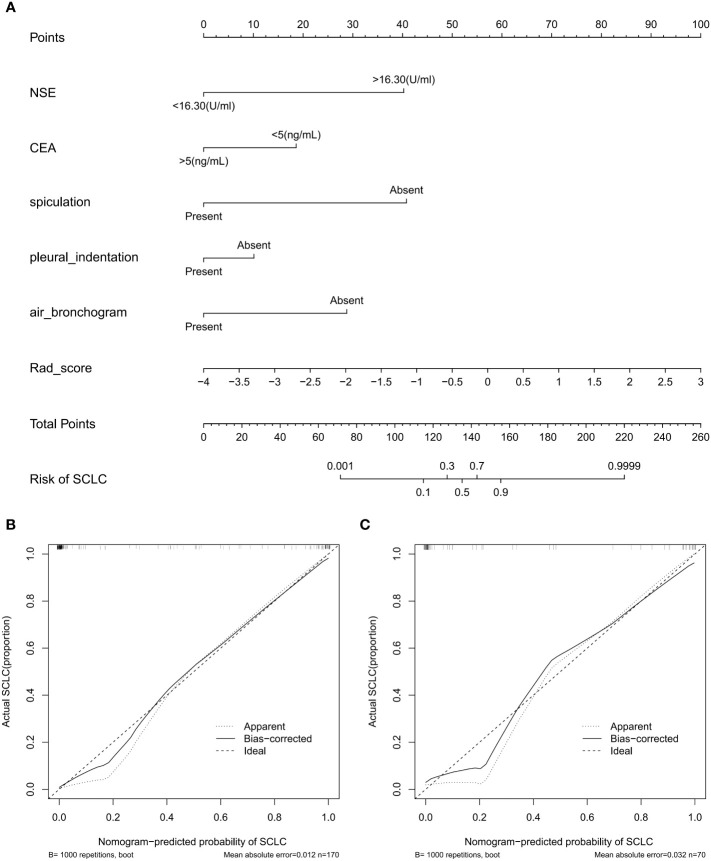
Radiomics-based nomogram **(A)** was developed in the training set, including the rad-score, NSE level, CEA level, spiculation, pleural indentation, and air bronchogram. Calibration curve of the nomogram in the training **(B)** and validation **(C)** datasets.

**Figure 6:**
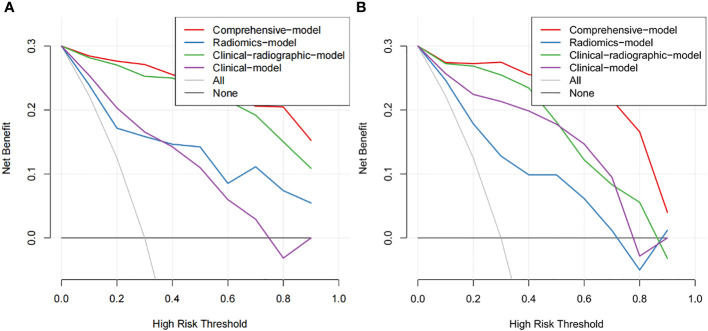
The DCA of the developed nomogram in training cohort **(A)** and validation cohort **(B)**. The y-axis and x-axis represent the net benefit and threshold probability, respectively. At the bottom, the black line “None” represented the assumption that none developed PSCLC, while the gray line “All” represented the assumption that all patients suffered PSCLC. The red line represented the net return of using the nomogram to predict PSCLC.

As shown in **Figure 5**, the nomogram was developed by combining rad-score, NSE level, CEA level, spiculation, pleural indentation, and air bronchogram. Calibration curves depicted the calibration of the nomogram in terms of the agreement between the predicted risk and the actual probability of PSCLC. The model showed favorable calibration in the validation set, with the Hosmer-Lemeshow test yielding a non-significant P=0.3748. **Figure 6** showed the DCA for the four models. The comprehensive model achieved more net clinical benefit than the other three models when differentiating between PSCLCs and PADCs in the training cohort (A) and the validation cohort (B).

## 4 Discussion

In the present study, we developed and validated a radiomics-based model nomogram that combined clinical data with subjective radiographic characteristics derived from CT images to help preoperatively distinguish PSCLC from PADC. The nomogram showed satisfactory diagnostic performance compared to the radiomics and clinical-radiographic models, with an AUC of 0.9851 for the training set and 0.9671 for the validation set. The comprehensive model incorporated serum tumor markers, CT signs, and the radiomics signature, further improving the diagnostic value.

Serum tumor biomarkers such as CEA, NSE, and CYFRA 21-1 have been revealed to be associated with several clinical events in lung cancer, including diagnosis of tumor subtypes, prognosis, and detection of tumor recurrence, all of which are closely associated with tumor burden (24). As shown in the results of the present study, serum tumor biomarkers, including CEA and NSE, were significant predictors in patients with PSCLC. In patients with an abnormal NSE level, a higher NSE level would indicate an increased likelihood of SCLC diagnosis, while the CEA level was higher in ADC than that in SCLC, which is consistent with previous studies (25). This study not only included serum tumor markers to explore the predictive markers but also considered the impact of other clinical factors such as smoking history, gender, and age on SCLC. SCLC is strongly associated with cigarette smoking (26, 27), but it was not found in the present study. This may be because traditional Chinese women smoke less. The inclusion of a small number of Chinese women in this study may lead to the possibility of sample selection bias.

Histopathological biopsy is the gold standard for accurate sub-classification of lung cancer. Peripheral lung cancer is mainly obtained by CT-guided percutaneous puncture lung biopsy, which is a complex invasive procedure, and the personal experience is a major determinant of procedural success (28). Sputum cytology is a convenient and non-invasive way to screen and diagnose lung cancer (29). However, this approach often gives negative results in the case of peripherally-placed cancers, although histopathology revealed positive diagnoses. CT screening for lung cancer has relatively high sensitivity and is the imaging modality of choice for diagnosing lung cancer (30, 31). In this study, the models only with clinical features achieved AUCs of 0.851 and 0.896 in the training and validation sets, respectively, which were lower than the comprehensive and clinical-radiographic models, showing the benefits of CT screening. Clinical symptoms of nodular lesions in the lung may appear later than imaging symptoms. Several recent studies have attempted to correlate different CT features of peripheral lung cancer with specific pathological subtypes (32, 33). Ground glass nodules (GGN) on chest CT could be a key imaging biomarker for early primary ADC in diagnosis, which had been observed at our institution (23, 34). Accordingly, mixed and pure GGN were excluded from this study. Both PSCLC and PADC can present as nodular lesions, and some CT signs may also be helpful for differential diagnosis (35). Many previous articles have studied the CT features of PSCLC. PSCLC tumors are often described as round or round-like nodules with lobulation, little spiculated signs, and internal necrosis. Due to its dense tumor cell arrangement with few fibrous tissues (36), air bronchogram were observed less frequently in PSCLC. In addition, pleural indentation was also uncommon in SCLC owing to the weak influence on the surrounding structure. Compared with that in PADC, spiculation was less common in PSCLC; these findings were consistent with previous results (37). SCLC typically presents as a large hilar mass and bulky mediastinal lymphadenopathy. It is uncommon for patients to present with a solitary peripheral nodule without central adenopathy (11). However, lymph node enlargement had no significant difference in the present study, which may be due to the selection bias caused by the relatively small sample.

Radiomics performs high-throughput extraction of quantitative image features, converts images into mineable data features, and subsequently analyses this data, providing valuable information for clinical decision-making (18, 21, 38). Junior et al. used radiomics-based CT features as well as machine learning models to differentiate various types of lung cancer, with AUCs yielding 0.97 and 0.71 at testing and validation, respectively, indicating the great potential of the method for differential diagnosis of lung cancer subtypes (39). In exploring the clinical value of radiomics features for histological subtypes of tumors such as SCLC and NSCLC, Liu et al. found that the radiomics-based nomogram combined with clinical factors outperformed the simple application of the radiomics signature (30), confirming that the combination of radiomics and clinical data could improve the predictive performance. The nomogram developed by Liu had good predictive performance (training cohort, AUC: 0.985; validation cohort, AUC: 0.966).

The nomogram model we constructed had higher differential diagnostic efficacy (training cohort, AUC: 0.9851; validation cohort, AUC: 0.9671). One possible explanation could be that this research focused on peripheral solid nodules, making it relatively easy to outline the tumor on CT images, thus avoiding the hilum and main bronchi. Besides, the combination of radiomics, clinical factors, and CT signs may be another reason. This study constructed four models based on radiomics features, clinical data, clinical-radiographic factors, and combined radiomics-clinical-radiographic features. All four had good diagnostic ability in distinguishing peripheral SCLC from ADC, and the comprehensive model also had the best predictive efficacy compared with the other three models by the Delong test (P<0.05) in the validation set. This study developed an individual nomogram model to facilitate clinical decision-making by incorporating clinical data, radiographic characteristics, and radiomics features. The nomogram graphically, visually, and intuitively displayed the extent to which each factor contributes to the diagnosis of PSCLC, providing a non-invasive and individualized analysis method. Mohammadhadi Khorram et al. stated that radiomics was useful for predicting the early-stage NSCLC recurrence, progression, and recurrence free survival (40). Besides, previous study had elucidated the prognostic value of radiomics in SCLC patients scheduled for first-line chemotherapy (41). However, to the best of our knowledge, no previous study has investigated radiomics approaches in the prognosis of PSCLC. This will be an important issue to be explored in our future studies. SCLC typically shows intense uptake on positron emission tomography (PET)/CT scans, reflecting its high metabolic activity (42). Metabolic parameters on pretreatment and posttreatment PET/CT scans can be used as a predictive marker of clinical outcome of SCLC (42). PET images may contain added valuable information, which would be explored in our future work.

One limitation of this study was the sample size of the retrospective single-center study, whose relatively small number of patients may have led to bias in the validation set. All high-resolution CT images in this study were obtained through the hospital’s picture archive and communication system (PACS). However, before 2017, the hospital did not save CT thin layer images. Therefore, we only included data between October 2017 to March 2022. Secondly, the paired controls enrolled in the present study were only from peripheral lung adenocarcinoma, while other pathological types of peripheral lung cancer were not included in this study due to their small number. We may need larger cohorts from other centers for further external cross-validation in later studies. Finally, this study only extracted the radiomics features from plain CT images. It may be worthwhile to expand the analysis to include contrast-enhanced CT and PET/CT images as part of the study in future research.

In conclusion, the differentiated clinical-radiographic model constructed in this study performed well in distinguishing PSCLC from PADC. On the basis of this, the nomogram developed based on clinical-radiographic factors combined with CT radiomics further improved the accuracy of diagnosing PSCLC and PADC, which was objective, non-invasive, and reproducible.

## Data availability statement

The raw data supporting the conclusions of this article will be made available by the authors, without undue reservation.

## Ethics statement

Written informed consent was obtained from the individual(s) for the publication of any potentially identifiable images or data included in this article.

## Author contributions

JW and FZ: Conception of the project, design of the study, analysis of the data, and writing of the article. YL, TG, and FX: Collection and processing of data. TL, XD and JW: Collection of data. ML: Critical revision of the manuscript. All authors contributed to the article and approved the submitted version.
