# Supplementary material for: CT radiomics model combined with clinical and radiographic features for discriminating peripheral small cell lung cancer from peripheral lung adenocarcinoma
Source: Front Oncol. 2023 Mar 20;13:1157891. doi: 10.3389/fonc.2023.1157891 (PMC10069670; doi:10.3389/fonc.2023.1157891)
Supplement: Supplementary file 1 [file DataSheet_1.doc]

**Supplementary Material**

**1. Radiomics Features**

Radiomics features extraction was applied to the CT images using pyradiomics procedures (<https://pyradiomics.readthedocs.io/en/latest/>) inserted in AK software (Artificial intelligence Kit; GE Healthcare). Original radiomics features contained the classes of First order statistics, Shape, and texture features while the texture features contained Gray level co-occurrence matrix (GLCM), Gray level Run-length matrix (GLRLM), Gray level size zone matrix (GLSZM), Neighboring Gray Tone Difference Matrix (NGTDM) and [Gray Level Dependence Matrix](https://www.abbreviationfinder.org/cn/acronyms/gldm_gray-level-dependence-matrix.html) (GLDM)**.** The details of each radiomics feature could be found in the pyradiomics docs. (<https://pyradiomics.readthedocs.io/en/latest/features.html>). Besides, we also extracted the high order statistics features: first processed the original images using different filters or mathematical transformations, then extracted their first order statistics and texture features. Eight filters were adopted in this study: Wavelet, LoG, Square, SquareRoot, Logarithm, Exponential, Gradient, and LocalBinaryPatern3D. Finally, a total of 1316 radiomics features were extracted from one single VOI in the images (Table S1)**.**

**Table S1 Summary of radiomics features used in this study**

| Feature classes | No. of features | 3 representative features |
| --- | --- | --- |
| First Order Statistics | 252 | RootMeanSquared, Maximum, MeanAbsoluteDeviation,… |
| Shape-based (3D) | 14 | VoxelVolume, SurfaceVolumeRatio, Maximum3DDiameter,… |
| GLCM | 336 | ClusterProminence, ClusterShade, ClusterTendency,… |
| GLRLM | 224 | ShortRunEmphasis, GrayLevelNonUniformity, GrayLevelNonUniformityNormalized,… |
| GLSZM | 224 | GrayLevelNonUniformity, ZonePercentage, GrayLevelVariance,… |
| NGTDM | 70 | Contrast, Coarseness, Strength,… |
| GLDM | 196 | DependenceNonUniformity, LowGrayLevelEmphasis, LargeDependenceHighGrayLevelEmphasis,… |
| Total | 1316 |  |

GLCM=Gray level co-occurrence matrix, GLRLM=Gray level Run-length matrix, GLSZM=Gray level size zone matrix, NGTDM=Neighboring Gray Tone Difference Matrix, GLDM=[Gray Level Dependence Matrix](https://www.abbreviationfinder.org/cn/acronyms/gldm_gray-level-dependence-matrix.html)

**2.**

**Table S2A. Inter-observer segmentation variability**

| 1 | 0.932865 |
| --- | --- |
| 2 | 0.799338 |
| 3 | 0.772356 |
| 4 | 0.861672 |
| 5 | 0.910718 |
| 6 | 0.898898 |
| 7 | 0.949083 |
| 8 | 0.890617 |
| 9 | 0.905234 |
| 10 | 0.921482 |
| 11 | 0.795324 |
| 12 | 0.901955 |
| 13 | 0.930109 |
| 14 | 0.876586 |
| 15 | 0.897615 |
| 16 | 0.851389 |
| 17 | 0.867561 |
| 18 | 0.860389 |
| 19 | 0.852825 |
| 20 | 0.779049 |

**Table S2B. Intra-observer segmentation variability**

| 1 | 0.882391 |
| --- | --- |
| 2 | 0.812888 |
| 3 | 0.901511 |
| 4 | 0.872586 |
| 5 | 0.915548 |
| 6 | 0.884448 |
| 7 | 0.784320 |
| 8 | 0.900482 |
| 9 | 0.778353 |
| 10 | 0.827651 |
| 11 | 0.790129 |
| 12 | 0.947480 |
| 13 | 0.912444 |
| 14 | 0.828414 |
| 15 | 0.898991 |
| 16 | 0.913771 |
| 17 | 0.932565 |
| 18 | 0.927737 |
| 19 | 0.756599 |
| 20 | 0.822710 |

1. **The rad-score calculation formula**

Rad-score=0.945723398-0.006840838× Wavelet-LLL_firstorder_90Percentile -0.126750926× Lbp-3D-m1_glszm_GrayLevelNonUniformity -0.013489915× Wavelet-LLH_glrlm_LongRunHighGrayLevelEmphasis -0.106548305× Wavelet-LHH_glszm_ZoneVariance +0.066670800× Wavelet-LLH_glcm_Correlation -0.034473688× Wavelet-LLL_firstorder_Kurtosis -0.303971900× Wavelet-LHL_glszm_GrayLevelNonUniformityNormalized +0.225560438× Wavelet-HLH_firstorder_Median +0.397117163× lbp-3D-k_firstorder_10Percentile +0.067134547× Log-sigma-3-0-mm-3D_firstorder_Skewness -0.027054686× Wavelet-HLH_glcm_InverseVariance +0.213892298× Lbp-3D-m2_glszm_SizeZoneNonUniformityNormalized +0.182694470× Wavelet-HHH_firstorder_Skewness +0.131469705× Log-sigma-3-0-mm-3D_firstorder_Maximum -0.264313730× Wavelet-HLL_glcm_MCC+0.200666178× Original_shape_Flatness +0.262056072× Wavelet-LHH_glszm_SmallAreaEmphasis +0.196563880× Wavelet-LHL_gldm_DependenceEntropy +0.147958576× Lbp-3D-k_firstorder_Skewness -0.050848959× Wavelet-HHL_glszm_SmallAreaLowGrayLevelEmphasis+0.169264790× Log-sigma-3-0-mm-3D_glcm_ClusterShade -0.114796398× Log-sigma-2-0-mm-3D_ngtdm_Strength -0.039928213× Wavelet-HHH_glszm_LargeAreaHighGrayLevelEmphasis.
